# POTENTIAL IMPLICATIONS OF ASPREE ON ASPIRIN PRIMARY PREVENTION GUIDELINES

**DOI:** 10.1093/geroni/igz038.2360

**Published:** 2019-11-08

**Authors:** Anne M Murray

**Affiliations:** Berman Center for Outcomes and Clinical Research, Minneapolis, Minnesota, United States

## Abstract

The 2016 USPSTF guidelines for aspirin to prevent CVD and colorectal cancer noted insufficient evidence to assess the balance of benefit and harm in those 70 and older. The long-awaited ASPREE trial, conducted in healthy elderly aged 70 and older (65 for US minorities), evaluated aspirin’s effect on disability-free survival, a composite of death, dementia, or persistent physical disability. CVD and cancer were pre-specified secondary endpoints, positioning ASPREE’s results to substantially inform the evidence gap noted in the USPSTF guidelines. Low-dose aspirin over 5 years did not lower CVD events or colorectal risk, but significantly increased bleeding. The ASPREE-XT observational follow-up study over the next 5-7 years will observe for potential legacy effects of aspirin on the primary and secondary outcomes of ASPREE, thus adding further evidence to define the risk-benefit profile of aspirin for primary prevention in healthy elderly.

